# Association between preoperative toe perfusion index and maternal core temperature decrease during cesarean delivery under spinal anesthesia: a prospective cohort study

**DOI:** 10.1186/s12871-021-01470-y

**Published:** 2021-10-21

**Authors:** Shohei Kaneko, Kentaro Hara, Shuntaro Sato, Takaya Nakashima, Yurika Kawazoe, Miyako Taguchi, Shigehiko Urabe, Akiha Nakao, Kozue Hamada, Michiko Yamaguchi, Tetsuya Hara

**Affiliations:** 1 Department of Anesthesia, National Hospital Organization Nagasaki Medical Center, 2-1001-1 Kubara, Omura, Nagasaki 856-8562 Japan; 2 Department of Anesthesiology and Intensive Care Medicine, Nagasaki University Graduate School of Biomedical Sciences, 1-7-1 Sakamoto, Nagasaki, Nagasaki 852-8501 Japan; 3 Surgery Center, National Hospital Organization Nagasaki Medical Center, 2-1001-1 Kubara, Omura, Nagasaki 856-8562 Japan; 4 Department of Health Sciences, Nagasaki University Graduate School of Biomedical Sciences, 1-7-1 Sakamoto, Nagasaki, Nagasaki 852-8520 Japan; 5 Clinical Research Center, Nagasaki University Hospital, 1-7-1 Sakamoto, Nagasaki, Nagasaki 852-8501 Japan; 6 Nagasaki University School of Medicine, 1-12-4 Sakamoto, Nagasaki, Nagasaki 852-8523 Japan

**Keywords:** Toe perfusion index, Parturient, Cesarean delivery, Core temperature, Perioperative hypothermia, Spinal anesthesia

## Abstract

**Background:**

The main mechanism of body temperature decrease during cesarean delivery under spinal anesthesia is core-to-peripheral redistribution of body heat, attributable to vasodilation. Perfusion index (PI) obtained with a pulse oximeter helps to assess peripheral perfusion dynamics by detecting the change in peripheral vascular tone. This study aimed to examine whether preoperative toe PI could predict the decrease in core temperature induced by spinal anesthesia during cesarean delivery.

**Methods:**

Parturients undergoing scheduled cesarean delivery under combined spinal-epidural anesthesia from September 2019 to March 2020 were enrolled in this single-center prospective cohort study. All parturients received 0.5% hyperbaric bupivacaine (10 mg) with fentanyl (15 μg) intrathecally. A pulse oximeter probe was placed on the left second toe for continuous PI measurement. The 3 M™ Bair Hugger™ Temperature Monitoring System placed over the right temporal region was used to record core temperature over time. We evaluated the association between the maximum core temperature decrease, which is the primary outcome, and the preoperative toe PI at operating room (OR) admission using a segmented regression model (SRM) and a generalized additive model (GAM). The maximum core temperature decrease was defined as the difference between core temperature at OR admission and minimum intraoperative core temperature.

**Results:**

Forty-eight patients were evaluated. In the SRM, the slope for the association between the maximum core temperature decrease and the preoperative toe PI changed from 0.031 to 0.124 after PI = 2.4%. Likewise, with the GAM, there was a small core temperature decrease when preoperative toe PI was greater than 2.0 to 3.0%.

**Conclusions:**

Low preoperative toe PI was associated with maternal core temperature decrease during cesarean delivery under spinal anesthesia. Preoperative toe PI is a simple, non-invasive, and effective tool for the early prediction of perioperative core temperature decrease during cesarean delivery.

**Trial registration:**

UMIN Clinical Trials Registry (registry number: UMIN000037965).

## Background

Neuraxial (spinal, epidural, or combined spinal-epidural technique) anesthesia is currently the anesthetic technique of choice for cesarean delivery. Spinal and epidural anesthesia cause body heat redistribution by vasodilation below the level of neuraxial sensory blockade [1]. Additionally, neuraxial techniques decrease the vasoconstriction and shivering thresholds even above the level of the sensory block, and directly block the efferent nerves that control vasoconstriction and shivering in the lower body [1]. Perioperative hypothermia (< 36.0 °C) has been estimated to occur in more than 60% of parturients undergoing cesarean delivery [2] and should be avoided because it generally contributes to serious complications such as coagulopathy, wound infections, myocardial ischemia, shivering, and patient discomfort [3–6].

Active warming of parturient during cesarean delivery reduces perioperative maternal hypothermia and shivering [7]. However, the benefits of single active warming on maternal temperature are limited, with most studies reporting no more than 0.2–0.5 °C core temperature difference between active warming and control groups [2]. Recently, several clinical studies have been conducted to preoperatively identify parturients at high risk of perioperative body temperature decrease and hypothermia during cesarean delivery; however, the results remain unclear [8].

Perfusion index (PI) obtained with a pulse oximeter is calculated as the ratio of pulsatile blood flow to non-pulsatile blood in the peripheral tissues [9]. It can be measured continuously and non-invasively and helps in the assessment of peripheral perfusion dynamics by detecting the change in peripheral vascular tone. The PI value varies dramatically from 0.02 to 20% and correlates with the change in blood flow at the monitored site. Low PI usually reflects peripheral vasoconstriction with or without severe hypovolemia, and high PI usually reflects peripheral vasodilation. The change in peripheral PI is a rapid indicator of the change in peripheral perfusion. This change is related to the vascular status, sympathetic responses, and anesthetic effects [10, 11]. Additionally, the change in peripheral PI also reflects the change in core-to-peripheral temperature gradients [9, 12]. Although these gradients have been used as a measure of peripheral vasoconstriction [13], temperature gradients before the induction of anesthesia affect the magnitude of body heat redistribution by vasodilation following anesthetic administration [14–16]. A prospective observational pilot study demonstrated that low baseline peripheral PI was the most relevant factor for the development of intraoperative hypothermia under general anesthesia [17]; therefore, this suggests that preoperative peripheral PI may be useful in predicting the magnitude of redistributive hypothermia. To our knowledge, no previous reports have investigated the association between baseline peripheral PI and body temperature decrease during cesarean delivery under spinal anesthesia. We hypothesized that low preoperative toe PI is associated with maternal core temperature decrease during cesarean delivery under spinal anesthesia. Our study aimed to examine whether preoperative toe PI could predict the decrease in core temperature induced by spinal anesthesia during cesarean delivery.

## Methods

This single-center prospective cohort study was conducted at the National Hospital Organization Nagasaki Medical Center, Nagasaki, Japan. This study was approved by our institutional research ethics committee (approval number: 2019059) on 2nd September 2019 and follows the Declaration of Helsinki. The study was registered with UMIN Clinical Trials Registry (Trial registry number: UMIN000037965, registration date: 8th September 2019) before the onset of participant enrollment. Written informed consent was obtained from each participant before study participation. This manuscript adheres to the Strengthening the Reporting of Observational Studies in Epidemiology (STROBE) guidelines.

### Participant selection

The inclusion criteria included the American Society of Anesthesiologists (ASA) physical status classification I-II parturient, between 18 and 40 years of age, with term gestation (≥ 37 weeks), and scheduled cesarean delivery with combined spinal-epidural anesthesia. Exclusion criteria were as follows: unscheduled cesarean delivery, morbid obesity (body mass index [BMI] ≥ 40 kg/m^2^), preoperative hyperthermia (> 38 °C) or preoperative hypothermia (< 36 °C), cardiovascular or cerebrovascular disease, hypothyroidism or hyperthyroidism, history of anxiety disorder, difficulty in maintaining the supine position, and contraindication to spinal anesthesia. The study recruitment period was from 17th September 2019 to 9th March 2020.

### Study protocol

No parturient received any premedication. Each parturient was kept off solid food for at least 6 h, and off clear water for 2 h before spinal anesthesia. A 20-gauge peripheral intravenous cannula was inserted at the obstetric ward. Room temperature Ringer’s lactate solution was administered at a flow rate of 80 mL/h, about 2 h before entering the operating room (OR). All parturients were directly transported from the ward to the OR without preoperative active warming (e.g., wearing socks, using body warming blanket), and the OR temperature was maintained at 27 °C.

Each parturient was rapidly infused intravenously with 500 mL of 6% hydroxyethyl starches 130/0.4 (Voluven®; Fresenius Kabi, Tokyo, Japan) for hydration before spinal anesthesia. Thereafter, Ringer’s lactate solution was infused about 10 mL/kg/h until the end of the surgery. Their infusion fluids in the OR were kept warm preoperatively at 38 °C in the heat insulating cabinet. Standard monitoring was performed with an electrocardiogram, automated non-invasive arterial pressure measurement on the right arm, and finger pulse oximetry on the left index finger. Besides, the pulse oximeter probe (Masimo Rainbow SET Pulse CO-Oximeter Radical 7; Masimo Corp., Irvine, CA, USA) was placed on the left second toe for continuous monitoring of the toe PI. For core temperature measurement, we attached the 3 M™ Bair Hugger™ Temperature Monitoring System (3 M Company, St. Paul, MN, USA) over the right temporal region. This Food and Drug Administration-approved Temperature Monitoring System can measure core temperature by heating the skin sensor attached to the forehead and reaching thermal equilibrium between sensor temperature and core temperature [18]. The mean error in measurement accuracy of this device was found to be − 0.23 °C (95% limits of agreement of ± 0.82 °C) compared with pulmonary artery temperature [18].

All parturients received combined spinal-epidural anesthesia in the right lateral decubitus position. After inserting an epidural catheter at the T12-L1 or L1–2 vertebral interspace, spinal anesthesia was performed at the L2–3 or L3–4 vertebral interspace. A 25-gauge Quincke spinal needle was inserted into the subarachnoid space, and 10 mg (2.0 mL) of 0.5% hyperbaric bupivacaine (Marcain®; Aspen Japan, Tokyo, Japan) with 15 μg (0.3 mL) fentanyl (Fentanyl®; Janssen Pharmaceutical K.K., Tokyo, Japan) were administered intrathecally. Following the securing of the epidural catheter, each parturient was returned to the supine position with a 15° left lateral tilt to facilitate the left displacement of the uterus. The tilted position was returned to the horizontal supine position after the maternal hemodynamics stabilized. The sensory blockade level was checked after spinal injection using cold ice. If T4 sensory block level was not achieved, 2% lidocaine (Xylocaine® Injection Polyamp 2%; Aspen Japan, Tokyo, Japan) was administered through the epidural catheter in 5 mL increments until it was achieved. To prevent post-spinal hypotension, phenylephrine at 0.3 μg/kg/min was started immediately after the induction of spinal anesthesia. Once the systolic blood pressure (SBP) was less than 80 mmHg or there were symptoms consistent with hypotension (e.g., dyspnea, nausea, or vomiting) even without SBP < 80 mmHg, a bolus of 50 to 100 μg phenylephrine or 4 mg ephedrine was administered depending on the patient’s heart rate (HR). When the patient’s HR was less than 60 beats/min without the occurrence of post-spinal hypotension, a bolus of 0.5 mg atropine was given. When the patient’s SBP was stable, the continuous administration of phenylephrine was gradually reduced, and was terminated at the discretion of the anesthesiologist.

Since the start of the surgery, the patient’s upper body was warmed using a 3 M™ Bair Hugger™ multi-position upper body warming blanket (Model 622; 3 M Company, St. Paul, MN, USA) attached to a 3 M™ Bair Hugger™ warming unit (Model 675; 3 M Company, St. Paul, MN, USA) set to 38 °C. The OR temperature was changed from 27 °C to 24 °C after placing the newborn baby in the infant incubator.

### Measurements

Patient characteristics and baseline parameters were obtained from electronic medical and anesthetic records. Toe PI and core temperature were recorded at one-minute intervals from entering the OR until the end of the surgery. Preoperative toe PI was defined as the average PI value measured for 3 min in the horizontal supine position, immediately before right lateral decubitus repositioning. All parturients were instructed to remain motionless and rested during the preoperative toe PI measurement. To evaluate the redistribution of body temperature after spinal anesthesia, we investigated the maximum core temperature decrease in the perioperative period. The primary outcome was the maximum core temperature decrease. In this study, the perioperative period was defined as the time from entering the OR until the end of the surgery, and the maximum core temperature decrease was defined as the difference between the core temperature at OR admission and the minimum intraoperative core temperature. Moreover, we evaluated shivering severity and thermal comfort when leaving the OR. Shivering severity was assessed using the Bedside Shivering Assessment Scale: 0 = no shivering, 1 = shivering localized to the core and neck, 2 = shivering including the upper extremities, 3 = total body shivering [19]. Thermal comfort was measured using the American Society of Heating, Refrigerating and Air-Conditioning Engineers (ASHRAE) scale, which is a seven-point Likert scale: Hot (+ 3), Warm (+ 2), Slightly warm (+ 1), Neutral “just right” (0), Slightly cool (− 1), Cool (− 2), and Cold (− 3) [20].

Surgery time was defined as the time between the start of the surgery and the end of the wound closure. Total volume of intravenous fluids, total dose of cardiovascular drugs administered, and estimated blood loss were also recorded.

### Statistical analysis

Patient characteristics and surgical data are presented as median [interquartile range], or the number of patients (%). Patient body temperature and toe PI, and data related to the decrease in maternal body temperature are presented as mean ± standard deviation (SD), median [interquartile range], or the number of patients (%). The Shapiro–Wilk test was used to determine normality. The sample size was not calculated since this was an observational study, and all patients meeting the eligibility criteria during the study period were included.

To analyze whether there is a relationship between preoperative toe PI and decreased maximal core temperature, firstly, correlation analysis was performed. Secondly, a segmented regression model (SRM) and a generalized additive model (GAM) were used. We suspected that the relationship between preoperative toe PI and decreased maximal core temperature was nonlinear from the scatter plot; thus, we conducted analyses using SRM and GAM.

SRM (also called change-point regression) is a practical analysis if we expect to have several slopes between dependent and independent variables different from a simple linear regression. These slopes quantify the change in the relationship between the two variables. Points where the slope changes are called “change-points.” The change-point can be interpreted as a critical, safe, or threshold value beyond or below which desired effects occur and is important in decision making [21]. In this study, we considered that two slopes exist between the dependent and independent variables from the scatter plot. We pre-determined four expected preoperative toe PI change-points based on quartile ranges (first, second, and third quartiles) and mean, created four SRMs, and evaluated the Akaike’s Information Criterion (AIC) of each model to determine the best fit model. GAM provides a modeling approach that combines powerful statistical methods with interpretability, smooth functions, and flexibility. Although generalized linear models, such as simple regression analysis, can only express linear relationships, GAM can also express non-linear relationships while maintaining interpretability and flexibility using multiple smoothing functions (smoothers). To find the smoother that best fits the data, the choice of smoothing parameters—i.e., the parameters that control the smoothness of the predictive functions—is key as in SRM [22]. We created several models to find the optimal parameters in GAM. The results of each model were evaluated using root mean squared error (RMSE) to determine the model that best fit the data. SRM and GAM included covariates associated with maternal hypothermia, which are BMI and core temperature at OR admission [23].

Statistical significance was defined as *P* value < 0.05. All statistical analyses were performed with R version 3.6.3 (R Foundation for Statistical Computing, Vienna, Austria).

## Results

**Fig. 1:**
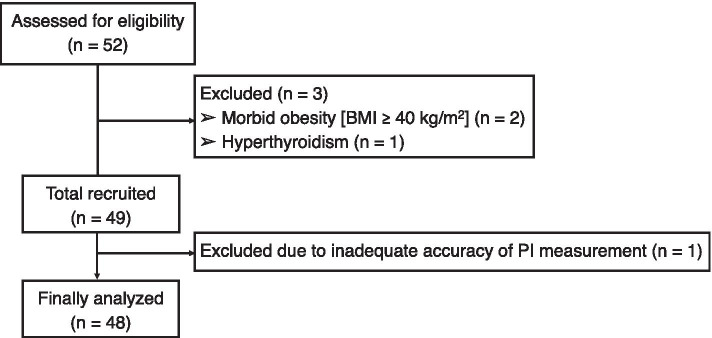
Flow diagram of patient enrollment and analysis in this study. BMI: body mass index; PI: perfusion index

A total of 52 patients were assessed for eligibility. Of those, three patients were excluded, resulting in the enrollment of 49 patients. One patient was excluded due to inadequate accuracy of PI measurement; 48 patients were finally evaluated. The flow diagram for excluded patients is shown in Fig. 1.

Patient characteristics are summarized in Table 1. No parturients began labor before surgery. Surgical characteristics and anesthesia management data are summarized in Table 2. The median [interquartile range] phenylephrine requirement was 620 [345 to 870] μg. Ephedrine was not administered to any parturient. The median [interquartile range] atropine requirement was 0 [0 to 0.5] mg.

**Table 1:** Patient characteristics

Age (years)	35 [31 to 37]
Height (cm)	159 [156 to 163]
Weight (kg)	65 [59 to 69]
BMI (kg/m^2^)	25.6 [22.8 to 27.5]
Gestational age (weeks)	38 [38 to 38]
Gravidity	3 [2 to 3]
Parity	1 [0 to 2]
Previous cesarean delivery	28 (58%)
Pregnancy-induced hypertension	2 (4%)
Gestational diabetes	2 (4%)
Twin pregnancy	7 (15%)

Data are presented as median [interquartile range], or the number of patients (%)

*BMI* body mass index

**Table 2:** Surgical characteristics and anesthesia management data

Surgery time (min)	76 [65 to 88]
Total volume of intravenous fluids (mL)	1423 [1252 to 1681]
Estimated blood loss (mL)	913 [696 to 1287]
Total dose of phenylephrine (μg)	620 [345 to 870]
Total dose of ephedrine (mg)	0 [0 to 0]
Total dose of atropine (mg)	0 [0 to 0.5]
Preoperative sensory block level (%)	
T2	11 (23%)
T4	37 (77%)
Number of epidural doses of 2% lidocaine (5 mL)	0 [0 to 0]
Sensory block level at end of surgery (%)	
T4	25 (52%)
T6	18 (38%)
T8	3 (6%)
T10	2 (4%)

Data are presented as median [interquartile range], or the number of patients (%)

**Fig. 2:**
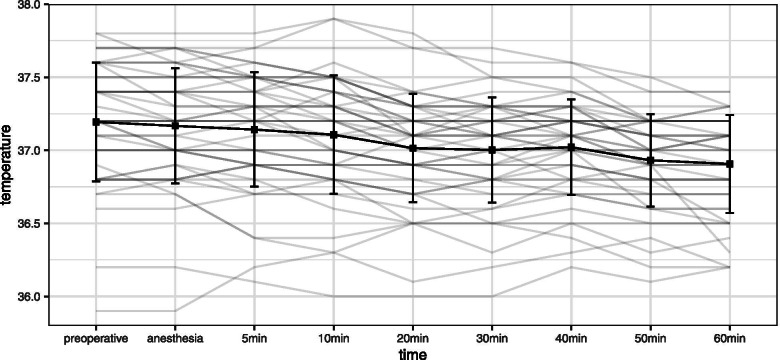
Parallel plot and mean ± SD of the core temperature. SD: standard deviation

Figure 2 shows a parallel plot, which represents the profile of an individual and the mean ± SD of the core temperature at each observation point. The core temperature tended to decrease gradually after spinal anesthesia. Data related to the decrease in maternal body temperature are listed in Table 3. The maximum core temperature decrease ranged from − 0.1 °C to − 1.1 °C, with a mean ± SD of − 0.4 ± 0.2 °C.

**Table 3:** Data related to the decrease in maternal body temperature

Minimum intraoperative core temperature	36.8 ± 0.4
Maximum core temperature decrease^a^ (°C)	−0.4 ± 0.2
Occurrence of intraoperative hypothermia (< 36.0 °C)	3 (6%)
Occurrence of shivering at leaving the OR	2 (4%)
Bedside Shivering Assessment Scale at leaving the OR	0 [0 to 0]
ASHRAE scale at leaving the OR	0 [−1 to 0]

Data are presented as mean ± SD, median [interquartile range], or the number of patients (%)

*ASHRAE scale* American Society of Heating, Refrigerating and Air-Conditioning Engineers scale, *OR* operating room, *SD* standard deviation

^a^Maximum core temperature decrease: the difference between the core temperature at OR admission and the minimum intraoperative core temperature

**Fig. 3:**
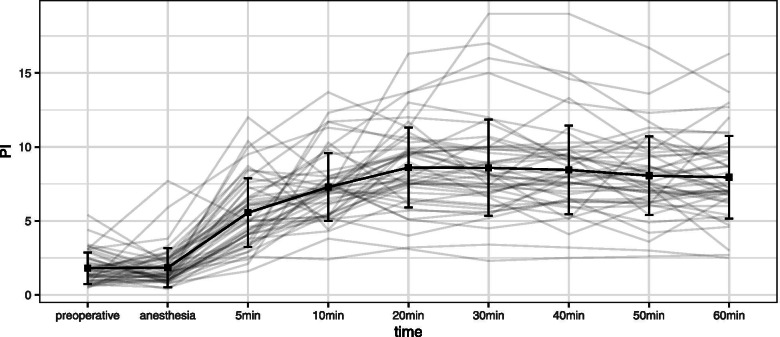
Parallel plot and mean ± SD of the toe PI. PI: perfusion index; SD: standard deviation

Figure 3 shows a parallel plot and the mean ± SD of the toe PI at each observation point. Preoperative toe PI ranged from 0.5 to 5.4%, with a mean ± SD of 1.8 ± 1.1%. The toe PI gradually increased after spinal anesthesia and did not change significantly after 20 min of anesthesia.

**Fig. 4:**
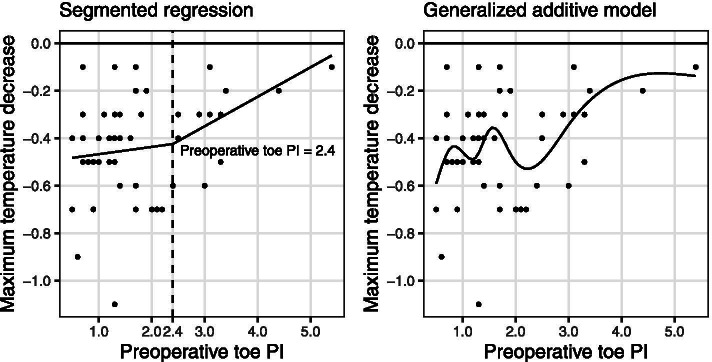
Scatter plots of the maximum core temperature decrease and the preoperative toe PI, and the predicted curve fitted by SRM and GAM. Maximum core temperature decrease: the difference between the core temperature at OR admission and the minimum intraoperative core temperature. SRM: segmented regression model; GAM: generalized additive model; OR: operating room; PI: perfusion index

Figure 4 shows the scatter plots of the maximum core temperature decrease and the preoperative toe PI, and the predicted curve fitted by SRM and GAM. In the SRM, the slope for the association between the maximum core temperature decrease and the preoperative toe PI, regression coefficients, changed from 0.031 to 0.124 after PI = 2.4%. In the GAM, there was a small decrease in core temperature when preoperative toe PI was greater than 2.0 to 3.0%.

## Discussion

In this prospective cohort study, we demonstrated that low preoperative toe PI was associated with the decrease in core temperature induced by spinal anesthesia during cesarean delivery. To the best of our knowledge, this study is the first to identify preoperatively parturients at high risk of intraoperative core temperature decrease during cesarean delivery.

The results of this study indicate that peripheral perfusion should be considered in the perioperative management of body temperature for parturients undergoing cesarean delivery under spinal anesthesia. The main mechanism of body temperature decrease in the early phase after spinal anesthesia is core-to-peripheral redistribution of body heat, just as during general anesthesia [1]. Spinal anesthesia causes the redistribution of body temperature by vasodilation below the level of neuraxial sensory blockade. The magnitude of this redistribution is affected by the temperature gradient between the core and peripheral thermal compartments before the induction of spinal anesthesia. This core-to-peripheral temperature gradient is maintained by thermoregulatory vasoconstriction; in particular, the vascular response is remarkable at the acral regions (e.g., fingers, toes, or nose), which have well-developed arteriovenous shunts and counter-current heat exchange mechanisms. Low peripheral perfusion due to vasoconstriction results in low peripheral temperature, which may lead to a decrease in core temperature after spinal anesthesia. Peripheral PI reflects perfusion changes associated with the thermoregulatory vascular responses, and the PI correlates with the core-to-peripheral temperature gradients [9, 12]. Therefore, patients with low peripheral PI have a greater redistribution of body temperature after the induction of anesthesia due to low peripheral temperature, resulting in core temperature decrease. Some researchers reported that low baseline peripheral PI was the most relevant factor in the development of intraoperative hypothermia under general anesthesia [17]. Their study results are similar to our findings that low preoperative toe PI can be a risk for intraoperative hypothermia in parturients undergoing cesarean delivery under spinal anesthesia.

Several previous studies have demonstrated that toe PI during cesarean delivery under spinal anesthesia correlates with the post-anesthesia blood pressure and partial pressure of oxygen in the umbilical vein [24, 25]; however, no reports have investigated the association between the baseline peripheral PI and body temperature decrease. We selected toe as the site of PI measurement in this study for two reasons: first, the toes are the prominent site for thermoregulatory vascular responses; second, the core temperature decrease in the early phase after spinal anesthesia is mainly attributed to the redistribution of body temperature from the core thermal compartment to the distal legs (lower leg and foot), which is greater than its redistribution to the proximal legs [26]. In the present study, toe PI also increased significantly after the induction of spinal anesthesia, which probably reflects increased toe perfusion due to the blood flow shift from the core compartment. Therefore, preoperative toe PI in parturients undergoing cesarean delivery under spinal anesthesia may be suitable for identifying those at high risk of intraoperative core temperature decrease. On the other hand, a previous secondary analysis in a randomized controlled trial showed that preoperative anterior thigh temperature does not correlate with the maximum perioperative temporal temperature decrease during cesarean delivery under spinal anesthesia [8]. This may have been influenced by the choice of anterior thigh temperature as peripheral temperature measurement site, where the magnitude of body temperature redistribution is small. Thus, anterior thigh temperature might not be reliable for measuring peripheral temperature related to core-to-peripheral temperature gradients.

In the present study, the maximum intraoperative core temperature decrease was − 0.4 ± 0.2 °C, which was within normal physiological variation in temperature (about − 0.5 °C) [27]. This decrease was small compared to the results of a previous observational study [7, 28], and this probably influenced the low incidence of maternal intraoperative hypothermia (< 36.0 °C) and shivering. The main reasons for this small decrease were maintenance of a relatively higher operating room temperature in our study [28, 29], continuous administration of phenylephrine after spinal anesthesia, and the omission of neuraxial hydrophilic opioids. Continuous phenylephrine administration suppresses the redistributive hypothermia after spinal anesthesia without contracting the arteriovenous shunts [30]. Additionally, a randomized double-blind controlled study showed that intrathecal morphine administration may exacerbate hypothermia [31]. However, in this study, preoperative toe PI was significantly associated with the magnitude of maternal core temperature decrease, despite the small decrease in the core temperature. It is necessary to confirm whether similar results as obtained by our study can be reproduced in parturients with a larger decrease in the core temperature.

The early identification of parturients at high risk of intraoperative core temperature decrease is desirable to prevent maternal hypothermia in the perioperative period of cesarean delivery under spinal anesthesia [8, 32]. Herein, our study results suggest that low preoperative toe PI may be an effective tool for the early prediction of maternal core temperature decrease during cesarean delivery. Furthermore, in parturients with low preoperative toe PI, preoperative active warming, which has been effective in patients undergoing cesarean delivery under spinal anesthesia, may have a greater effect on preventing perioperative maternal hypothermia [33]; however, it remains unclear which parturients benefit more. In general, preoperative active warming aims to prevent the redistribution of body temperature by increasing the peripheral temperature and decreasing the core-to-peripheral temperature gradient before the induction of anesthesia. Parturients with low preoperative toe PI are expected to have low peripheral temperature and a large core-to peripheral temperature gradient; therefore, preoperative active warming may prevent perioperative maternal hypothermia. However, since no previous study has examined the efficacy of preoperative active warming considering the preoperative peripheral PI in perioperative temperature management, further investigation is needed to elucidate this topic.

This study has several limitations. Firstly, the incidence of maternal hypothermia in the study population was lower than in those of similar prospective studies, which may have limited our ability to detect major differences within the population. Accordingly, we have been unable to determine a specific preoperative toe PI cutoff value to predict maternal hypothermia. Secondly, we did not measure the toe temperature, owing to insufficient equipment. Therefore, we could not measure the changes in peripheral temperature and core-to-peripheral temperature gradients; thus, the association between these changes and toe PI changes in this study is unclear. Thirdly, this study had a small sample size and was conducted at a single institution. Another limitation is that peripheral PI values widely vary among individuals [13]. A large prospective cohort study is required to confirm whether the results of SRM and GAM in our study are useful for screening all parturients at high risk of intraoperative maternal temperature decrease.

## Conclusions

We demonstrated that low preoperative toe PI was associated with maternal core temperature decrease during cesarean delivery under spinal anesthesia. Preoperative toe PI is a simple, non-invasive, and effective tool for the early prediction of perioperative core temperature decrease during cesarean delivery; therefore, its efficacy and clinical application should be further evaluated by future studies.

## Data Availability

The datasets used and analysed during the current study are available from the corresponding author on reasonable request.

## Abbreviations

ASA: American Society of Anesthesiologists
ASHRAE: American Society of Heating, Refrigerating and Air-Conditioning Engineers
AIC: Akaike’s Information Criterion
BMI: Body mass index
GAM: Generalized additive model
HR: Heart rate
OR: Operating room
PI: Perfusion index
RMSE: Root mean squared error
SBP: Systolic blood pressure
SD: Standard deviation
SRM: Segmented regression model
STROBE: Strengthening the Reporting of Observational Studies in Epidemiology
